# Numerical Investigation of the Composite Action of Axially Compressed Concrete-Filled Circular Aluminum Alloy Tubular Stub Columns

**DOI:** 10.3390/ma14092435

**Published:** 2021-05-07

**Authors:** Faxing Ding, Changbin Liao, En Wang, Fei Lyu, Yunlong Xu, Yicen Liu, Yuan Feng, Zhihai Shang

**Affiliations:** 1 School of Civil Engineering, Central South University, Changsha 410075, China; dinfaxin@csu.edu.cn (F.D.); Changbinliao@163.com (C.L.); wangen718@csu.edu.cn (E.W.); xuyunlong@csu.edu.cn (Y.X.); liuyicen796@163.com (Y.L.); 2 Engineering Technology Research Center for Prefabricated Construction Industrialization of Hunan Province, Changsha 410075, China; 3 China Southwest Architectural Design & Research Institute Corp. Ltd., Chicago, IL 60603, USA; xnyfy@vip.163.com; 4 MCC Capital Engineering & Research Incorporation Limited, Beijing 100176, China; shangzhihai@csri.com.cn

**Keywords:** aluminum alloy, concrete-filled circular aluminum alloy tubular (CFCAT) stub columns, finite element (FE) analysis, ultimate bearing capacity, confinement effect

## Abstract

This paper studied the composite action of concrete-filled circular aluminum alloy tubular (CFCAT) stub columns under axial compression. A fine-meshed finite three-dimensional (3D) solid element model making use of a tri-axial plastic-damage constitutive model of concrete and elastoplastic constitutive model of aluminum alloy was established. A parametric study utilizing the verified finite element (FE) model was carried out and the analytical results were exploited to investigate the composite actions of concrete-filled circular aluminum alloy tubular stub columns subjected axial compression. Compared with the concrete-filled steel tube (CFCST) stub columns, the aluminum alloy tube exerted a weaker constraint effect on the infilled concrete due to its lower elastic modulus. Based on the FE analytical results and regression method, the composite action model of concrete-filled circular aluminum alloy tubular stub columns was proposed. By generalizing the stress nephogram of the concrete-filled circular aluminum alloy tubular stub column at the limit state, a design formula was proposed to estimate the ultimate bearing capacity the columns using the superposition method. The predicted results of the proposed formula show a good agreement with both the experimental and FE analytical results. The comparison between the proposed formula and current design methods indicates that the proposed formula is more accurate and convenient to use.

## 1. Introduction

Aluminum alloy has been gradually used in building structures because of its merits of lightweight, lustrous appearance, good corrosion resistance, good processing performance and easy regeneration as utilized in the Inter-America Exhibition Center of San Paulo (Brazil), the International Congress Centre of Rio de Janeiro (Brazil), the Sport Hall of Quito (Ecuador), the Memorial Pyramid in La Baie (Canada), the Shanghai Botanical Garden Exhibition Greenhouse (China) and the Zero Magnetic Laboratory of Beijing Aerospace Experimental Research Center (China) [1,2,3]. On the other hand, the aluminum alloy has a lower Young’s modulus (about one-third compared with carbon steel), yield and ultimate strength compared with carbon steel, which results in a lower bearing capacity and larger deformation when at sustain load that has hindered its ulterior promotion in the engineering practice. To make full use of its advantages and avoid its defects, researchers have recently put forward a concept of using aluminum alloy tubes as a substitution of the steel tubes in concrete-filled steel tubular columns (CFST) that has gained wide attention in the scientific community [4,5,6,7,8,9,10]. Extensive studies on the mechanical behaviors of CFST have been carried out both theoretically and experimentally [11,12,13]. Regarding the axial bearing capacity concerned in this paper, the confinement provided by the outer steel tube considerably improved the mechanical behaviors of the infilled concrete by delaying the crush and cracks, while the inward local buckling of steel tubes was prevented by the infilled concrete [14]. Such beneficial interactions under compressive loads in the CFST columns were expected and also exist in the concrete-filled aluminum alloy tubular (CFAT) columns, so the performance of the aluminum alloy tube is upgraded.

Since the mechanical properties of aluminum alloy are somehow different from the carbon steel, specialized studies on the axially loaded behaviors of CFAT columns have been conducted by various researchers [6,7,8,15,16]. Although it is still insufficient compared with CFST columns related research, some consensus has been reached by the scientific community such as on its excellent load-carrying capacity. For instance, a series of tests have been accomplished by Feng Zhou and Ben Young [6,7,8] and the ultimate bearing capacity, failure modes and the load-shortening curves of the square, rectangular [6], circular [7] and double-skin [8] CFAT stub columns were investigated. While the reliability of the American specification is known [17,18], the Australian and New Zealand standards [19,20] were also evaluated against the test results and the predicted strength of square and rectangular columns using current design codes were considered unconservative. On the basis of the aforementioned experimental study, a parametric study of concrete-filled circular aluminum alloy tubular (CFCAT) columns subjected to the axial load using a numerical approach was carried out [21,22] and a design equation with higher accuracy than current design codes was proposed. KZ Nasser [15] conducted axial compression tests of 24 CFCAT stub columns specimens as well and the influence of diameters, diameter-to-thickness ratio and slenderness ratio on the columns bearing capacity were discussed. In addition, a test of lightweight aggregate concrete-filled aluminum alloy tube was performed by Resan [16]. It was found that the combination of aluminum alloy tube and lightweight aggregate concrete can provide a remarkable high strength-to-weight ratio in the specific structural members. The existing experimental studies indicated that the axially loaded behaviors of CFAT stub columns are similar to that of conventional CFST stub columns. To further discuss the mechanism of composite interaction in the axial loaded CFAT columns, numerical study, especially the fine finite element (FE) model analysis was presented by a small number of scholars [22,23,24,25,26]. For example, Wang et al. [22] established a fine-meshed FE model utilizing the general software ABAQUS [23] with the consideration of the interaction between the aluminum alloy tube and infilled concrete. The multi-stress condition of the aluminum alloy tube was analyzed and parametric study based on the established model was conducted. Similar investigations were performed by other researchers including Zhao et al. [24], Idan [25] and Patel et al. [26] as well. To sum up, the aforementioned numerical studies are mainly focused on the discussion of the factors which affect the mechanical behaviors of CFAT columns under axial compression, but the investigations on the confinement effect and the composite action between the aluminum alloy tube and infilled concrete is still inadequate, and there is no specialized numerical model of the composite action of CFCAT columns has been reported.

The existing studies partially addressed the mechanical behaviors of CFAT stub columns under axial loading and supported its prospect of engineering application. However, more in-depth investigations are needed to thoroughly explain the difference between the confinement effect in the CFST and CFAT columns and to reflect this impact in the design formulas. In the meantime, the promotion of CFAT columns usage in structural engineering requires practical, simple yet accurate design tools. However, up to now, the specific calculation method of ultimate bearing capacity of CFAT columns is still rare and their derivation is very complicated or without clear physical meanings.

Hence, the main purpose of the presented study is to numerically evaluate the discrepancy of the composite action in CFCST and CFCAT stub columns and to propose an accurate yet concise design formula of axial bearing capacity of CFCAT stub columns. The main contents of this paper include: (1) A 3D FE model of CFCAT stub columns under axial compression was established making use of the tri-axial plastic-damage constitutive model of confined concrete and the elastoplastic constitutive model of the aluminum alloy. The validity of the established FE model was verified against the collected test results. Then, the non-linear FE analysis was carried out to simulate the entire axial loading process of CFCAT stub columns. (2) A parametric study was performed, the difference of composite action between the CFCAT stub columns and CFCST stub columns were discussed. A composite action model of CFCAT stub columns was proposed. (3) A concise design formula incorporating an enhancement factor considering the equilibrium conditions in the ultimate state was derived. The proposed composite action model was utilized to derivate the design formula. The proposed formula was assessed more accurate and concise than the existing design formulas.

This paper is organized as follows. CFCAT stub columns FE modeling, the selecting of constitutive models and the model validation are presented in Section 2. The numerical investigation of CFCAT stub columns subjected axial loading and the influence is presented in Section 3. Section 4 simply introduces the composite action model of CFCAT stub columns. Section 5 presents the practical design formula for axial load-bearing capacity of CFCAT stub columns.

## 2. FE Model of CFCAT Stub Columns

### 2.1. Modeling of CFCAT Stub Columns

#### 2.1.1. Mesh Size and Element Type

**Figure 1:**
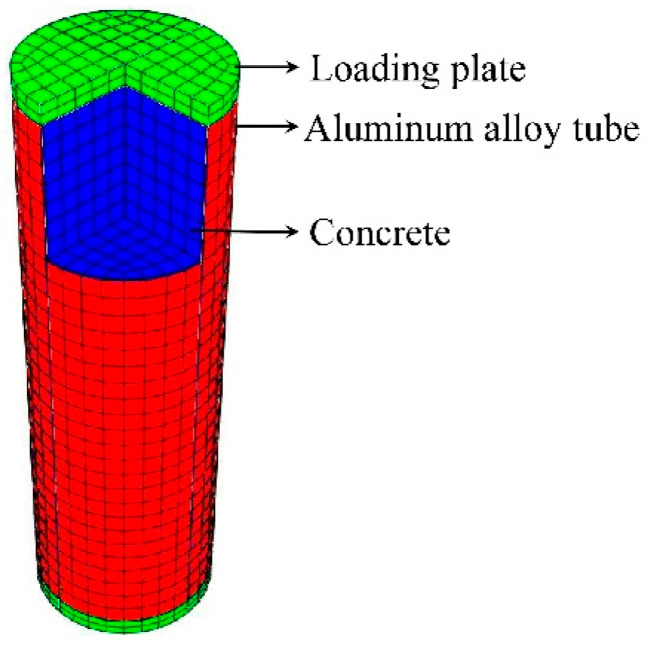
FE model of CFCAT stub column.

The 3D FE model of CFCAT stub column was established utilizing finite element analysis software ABAQUS version 6.14 [23]. The 8-node reduced integral format 3D solid element (C3D8R) was applied to simulate the aluminum alloy tube, infilled concrete and the loading plate. The structured meshing technique available in the ABAQUS was adopted and a convergence check of mesh size was conducted to ensure the accuracy and efficiency of performed numerical study. The mesh size suggested by Zhou and Young [21] that the length-to-width-to-depth ratio of each element around 1:1:1 was reported rational and effective and was adopted in this study. According to Liu [27], considering the accuracy of the prediction results and the computation time of the model, the mesh size of *D*/10 is rational for FE models, where *D* is the outside diameter of the aluminum alloy tube. The typical meshed size of the entire model is shown in Figure 1.

#### 2.1.2. Loading and Boundary Conditions

**Figure 2:**
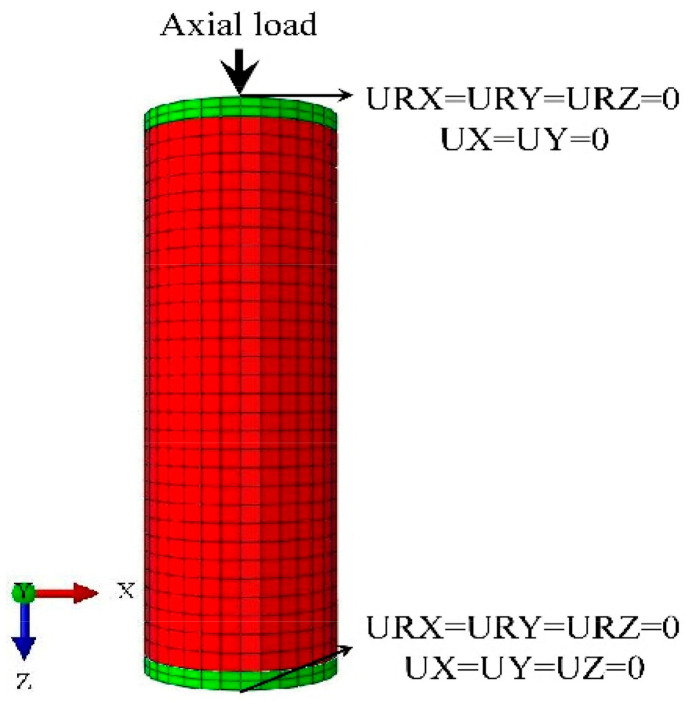
Boundary conditions of the FE models.

The axial load was applied at the center of the loading plate in the manner of displacement control on the top to simulate the axial loading process. The bottom ends of the stub columns were fixed against all degrees of freedom, the top end of the stub columns were fixed three directions of rotation and two horizontal directions of displacement while releasing the vertical displacement, as shown in Figure 2. Both material and geometric nonlinearities were taken into consideration and were solved by the incremental iteration method.

#### 2.1.3. Interaction of the Aluminum Alloy Tubes and the Infilled Concrete

The surface-to-surface contact pairs were adopted for the interaction between the aluminum alloy tube and the infilled concrete. The inner surface of the aluminum alloy tube was selected as the salve surface and the external surface of infilled concrete was chosen as the master surface [21]. Limited-slip was selected in the sliding formulation while the discretization method was surface-to-surface. The tangential behavior and normal behavior of the contact pair were defined as the contact property, while the penalty function was applied to the friction formula for the tangential behavior, in which the friction coefficient was 0.25 [21]. The normal behavior was set to “hard” contact which allowed separation after the occurrence of contact. The connection between the top surfaces of the aluminum alloy tube, core concrete and the bottom surface of the loading plate were tie connection while the nodes on the loading plate were adopted as the master nodes. This is to make sure that the axial load was simultaneously applied to the aluminum tube and the infilled concrete during the entire test progress. The deformation of the loading plate was assumed small enough thereby can be neglected and therefore was simulated by the rigid body element with the elastic modulus of 1 × 10^12^ MPa and the Poisson’s ratio of 1 × 10^−7^.

### 2.2. Constitutive Models of Infilled Concrete

The concrete damage plasticity (CDP) model has the ability to simulate a large number of quasi-brittle materials [28,29].

A tri-axial plastic-damage constitutive model of concrete under axial compression proposed by Ding et al. [30] was adopted in the presented numerical study. The uniaxial strain–stress relation of infilled concrete can be expressed as:

(1) $$y=\left\{\begin{array}{ll} \frac{Ax+(B-1)x^{2}}{1+(A-2)x+Bx^{2}},\; & x\leq 1\\ \frac{x}{\alpha {\left(x-1\right)}^{2}+x},\; & x>1 \end{array}\right.$$

where *y* = *σ*/*f*_c_ and *x* = *ε*/*ε*_c_ are the stress and strain ratios of the infilled concrete under the uniaxial compression, respectively. *σ* and *ε* are the stress and the strain of the infilled concrete. *f*_c_ = 0.4*f*_cu_^7/6^ is the uniaxial compressive strength of concrete [30], where *f*_cu_ stands for the concrete cube compressive stress. *ε_c_* is the strain corresponding with the peak compressive stress of concrete, *ε*_c_ = 383 *f*_cu_^7/18^ × 10^−6^. *A* represents the ratio of the initial tangent modulus to the secant modulus at peak stress, *A* = 9.1 *f*_cu_^−4/9^. *B* = 1.6 (*A* − 1)^2^ is a parameter that controls the decrease in the elastic modulus along the ascending branch of the axial stress–strain relationship. When the steel ratio is more than 2%, parameter α is taken as 0.15 according to the previous study [30].

The tri-axial plastic-damage behaviors of infilled concrete was defined using the concrete damage plasticity (CDP) model: the eccentricity is taken as 0.1, the ratio of the initial equibiaxial compressive yield stress to the initial uniaxial compressive yield stress (*f*_b0_/*f*_c0_)is 1.225, the ratio of the second stress invariant on the tensile meridian to that on the compressive meridian is 2/3 [31], the viscosity parameter is 0.005, and the dilation angle is 40°. The abovementioned constitutive plastic-damage model of infilled concrete has been verified in the previous study of various sections of CFST stub columns under axial compression [30]. The constitutive model is not depend on the shape and material of the metal tube filled [32], therefore is applicable to simulate the CFCAT stub columns subjected axial compression.

The conversion formula of concrete cylinder strength *f*_c_’ and concrete cube strength *f*_cu_ used in the analysis of collected stub column specimens are taken as [33]:

(2) $${f^{\prime }}_{c}=\left\{\begin{array}{ll} 0.8f_{\mathrm{cu}},\; & f_{\mathrm{cu}}\leq 50\mathrm{MPa}\\ f_{\mathrm{cu}}-10\;,\; & f_{\mathrm{cu}}>50\mathrm{MPa} \end{array}\right.$$

### 2.3. Constitutive Models of Aluminum Alloy Tube

Referring to Gardner and Ashraf [34], the expression of the stress–strain relation of the aluminum alloy is taken as:

(3) $$\varepsilon =\left\{\begin{array}{l} \frac{\sigma }{E_{0}}+0.002{\left(\frac{\sigma }{\sigma_{0.2}}\right)}^{n},\;\sigma \leq \sigma_{0.2}\\ \frac{\sigma -\sigma_{0.2}}{E_{0.2}}+\left(0.008-\frac{\sigma_{1.0}-\sigma_{0.2}}{E_{0.2}}\right){\left(\frac{\sigma -\sigma_{0.2}}{\sigma_{1.0}-\sigma_{0.2}}\right)}^{{n^{\prime }}_{0.2,1.0}}+\varepsilon_{0.2},\;\sigma >\sigma_{0.2} \end{array}\right.$$

(4) $$E_{0.2}=\frac{E_{0}}{1+0.002n/\left(\sigma_{0.2}/E_{0}\right)}$$

where *E*_0_ is the elastic modulus, *E*_0.2_ is the tangent stiffness at the 0.2% proof stress; *σ*_0.2_ and *σ*_1.0_ are the 0.2% and 1% proof stress of the aluminum alloy, respectively. The ratio *σ*_1.0_/*σ*_0.2_ is taken as 1.08 for T4 temper material and 1.04 for the T6 and T7 tempers [34]. *n* and *n^’^*_0.2,1.0_ are the strain hardening exponents, *n^’^*_0.2,1.0_ is taken as 4.5 [34]. *ε*_0.2_ is the strain at *σ*_0.2_. According to Eurocode 9 [35], *E*_0_ of the aluminum alloy is taken as 70Gpa, Poisson’s ratio *ν* is taken as 0.3. The nominal stress–strain curves above are converted to the true stress-log plastic strain which is applicable for FE modeling analysis [36]:

(5) $$\sigma_{\mathrm{true}}=\sigma_{\mathrm{nom}}(1+\varepsilon_{\mathrm{nom}})$$

(6) $$\varepsilon_{\ln}^{\mathrm{pl}}=\ln(1+\varepsilon_{\mathrm{nom}})-\sigma_{\mathrm{true}}/E_{0}$$

where *σ*_true_ and *σ*_nom_ are the true stress and nominal stress of the aluminum alloy tubes, respectively. *ε*_nom_ and *ε*_ln_^pl^ are the nominal strain and log plastic strain, respectively.

As a comparison, an elastoplastic model, considering Von Mises yield criteria, the Prandtl–Reuss flow rule and isotropic strain hardening, was utilized to describe the constitutive relation of low carbon steel. The stress–strain relationship of steel [30] is:

(7) $$\sigma =\left\{\begin{array}{ll} E_{0}\varepsilon , & \varepsilon \leq \varepsilon_{y}\\ f_{y},\; & \varepsilon_{y}<\varepsilon \leq \varepsilon_{\mathrm{st}}\\ f_{y}+\zeta E_{0}(\varepsilon -\varepsilon_{\mathrm{st}}), & \varepsilon_{\mathrm{st}}<\varepsilon \leq \varepsilon_{u}\\ f_{u},\; & \varepsilon_{u}<\varepsilon \end{array}\right.$$

where *f*_y_ and *f*_u_ (*f*_u_ = 1.5*f*_y_) are the yield and ultimate strength of steel, respectively. *E*_0_ of steel is taken as 210Gpa. *ε*_y_, *ε*_st_, *ε*_u_ are the yield strain, hardening strain and ultimate strain of steel, respectively, in which *ε*_u_ = *ε*_st_ + 0.5*f*_y_/*ζE*_0_, *ε*_st_ = 12*ε*_y_, *ε*_u_ = 120*ε*_y_, *ζ* = 1/216.

**Figure 3:**
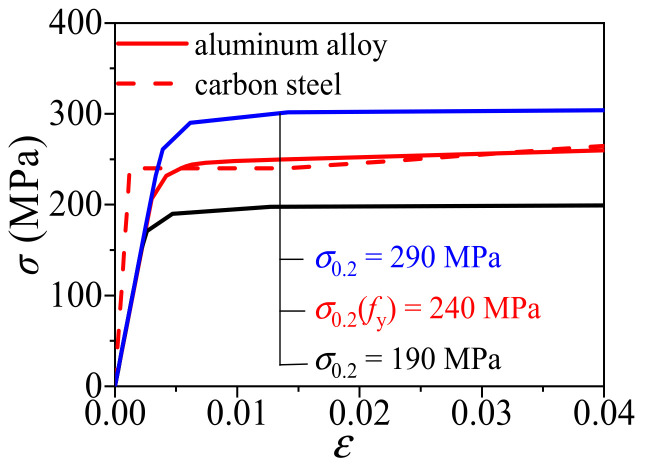
Comparison of the constitutive relationship between aluminum alloy and carbon steel.

Figure 3 shows the stress–strain relationship curves of the aluminum alloy and structural steel when their yield strengths are both 240MPa. It can be seen that the elastic modulus of the aluminum alloy is smaller than that of steel and there is no obvious yield platform as well.

### 2.4. Geometric Imperfection

The geometric imperfection effect was considered the non-linear numerical simulation of CFCAT stub columns under the compressive loading. The linear perturbation buckling analysis method is used to consider the elastic material properties of the aluminum alloy tubes separately. Firstly, a compressive displacement load (1 mm) is applied to the top of the aluminum alloy tube (model A), the eigenvalue buckling analysis is performed and the first mode is extracted. Secondly, model A is copied to model B, take the first mode of model A as the initial geometric imperfection of model B. The overall initial geometric imperfection factor is usually taken as 1/1000 of the length of the component. Finally, model B considers the overall initial geometric imperfection, and the compressive behavior analysis can be performed.

Residual stress appears in the aluminum alloy tubes on account of welding. The residual stress in the aluminum alloy section causes the initial stiffness of the whole columns to decrease slightly [37,38]. After irrigating concrete into the aluminum alloy tubes, the effect of residual stress is further reduced [39,40], which is negligible on the overall behavior of CFCAT. Consequently, the effect of residual stress was not taken into account in the finite element analysis.

### 2.5. Model Validation

**Figure 4:**
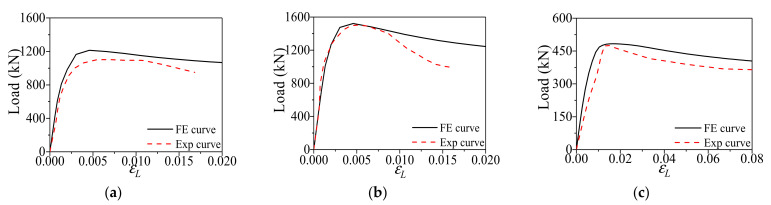
Comparison of FE and experimental load–strain curves: (**a**) test failure mode [7]; (**b**) aluminum failure mode; (**c**) infilled-concrete failure mode.

**Figure 5:**
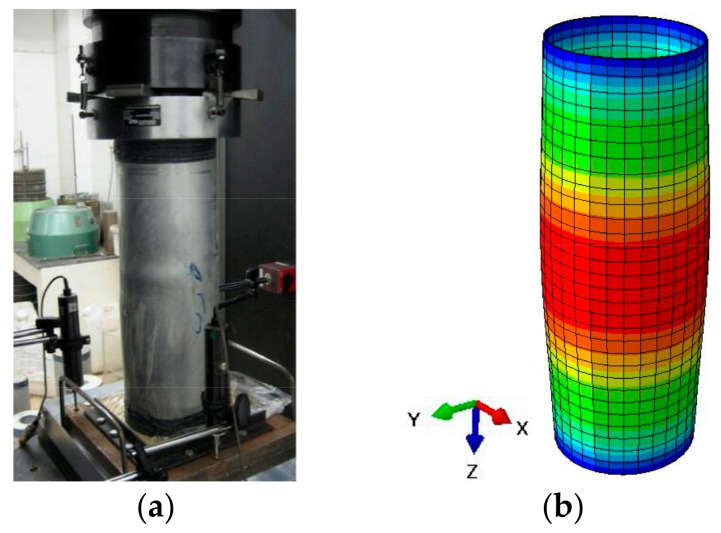
Comparison of experimental and FE models: (**a**) test failure mode [7]; (**b**) aluminum alloy tube failure mode.

The FE models of CFCAT stub columns under axial compression were verified against the experimental result presented by Zhou and Young [7] and Gong [41]. The ultimate bearing capacity calculated by the FE analysis and the experimental results were compared in Table 1. The average value of the ratios (*N*_u,exp_/*N*_u,FE_) is 0.996 and the coefficient of variation (COV) was 0.073. Meanwhile, the typical load–strain curves of FE simulation and the tests results were presented in Figure 4. It should be noticed here that the displacement of the specimen LV100-2 in Figure 4c was reported to be measured from the ends of the test machine which results in a small stiffness. To sum up, according to Table 1 and Figure 4, a generally good agreement was achieved between the FE analysis and the test results, especially for the prediction of ultimate bearing capacity. Figure 5 shows the comparison of the experimental and FE analysis failure modes. Thus, the established FE models can be utilized to carry out the further parametric study of the composite actions.

## 3. Numerical Investigation of CFCAT Stub Columns Subjected Axial Loading

### 3.1. Parametric Study

Based on the validated FE modeling approach, full-scale FE models were established to further investigate the mechanical behaviors of CFCAT stub columns under axial compression. The parameters analyzed herein were taken as: the constant diameter of *D* = 500 mm and the length of columns *L* = 1500 mm; the thickness of the aluminum alloy tube was taken as a variable of *t* = 5 mm, 10 mm, 15 mm, respectively and the aluminum ratios ρ range from 3.96% to 11.64%; the yielding strength of the aluminum alloy *σ*_0.2_ is 190 MPa, 240 MPa and 290 MPa respectively, while the concrete strength ranged from 40 MPa to 100 MPa. The temper of the aluminum alloy was taken as T4 uniformly. The strength of the aluminum alloy tube and the infilled concrete were paired according to the practical engineering application of the CFCAT stub columns: *σ*_0.2_ = 190 MPa was matched to *f*_cu_ = 40 MPa and 60 MPa, *σ*_0.2_ = 240 MPa was matched to *f*_cu_ = 60 MPa and 80 MPa, *σ*_0.2_ = 290 MPa was matched to *f*_cu_ = 80 MPa and 100 MPa. The parameters of established CFCAT stub column models were summarized in the Table 2. Where n is the strain-hardening exponent reflecting the degree of strain hardening of the aluminum alloy. The value of n was within a range from 23 to 31 according to Eurocode 9 [35]. Additionally, a FE model of CFCAT stub columns with *E*_0_ = 210 GPa was established as well for the comparison of CFCAT and CFCST stub columns.

**Figure 6:**
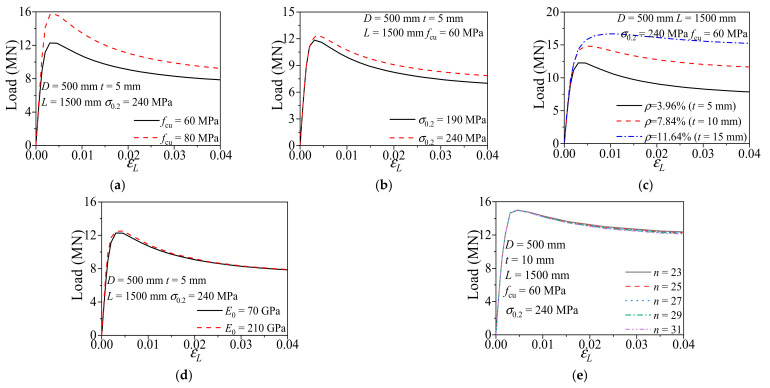
Influence of parameters on the ultimate bearing capacity: (**a**) concrete strength; (**b**) yielding strength; (**c**) steel ratio; (**d**) elastic modulus; (**e**) strain hardening exponent.

The typical *N*-*ε*_L_ curves obtained from the numerical study were presented in Figure 6. As shown in Figure 6a, when *f*_cu_ was increased from 60 MPa to 80 MPa, the ultimate bearing capacity of CFCAT stub columns was increased by 73.1%. And the stiffness of the specimen increased whereas the ductility decreased. Shown in Figure 6b, when *σ*_0.2_ increased from 190 MPa to 240 MPa, the ultimate bearing capacity was increased by 3.6%. The Figure 6c shows that when *ρ* increased from 3.96% to 7.84% and 11.64%, the ultimate bearing capacity was increased by 21% and 36%, respectively. When *E*_0_ increased from 70 GPa to 210 GPa, the ultimate bearing capacity was increased by 2% and elastic stiffness increased by 13%. Meanwhile, the ultimate bearing capacity almost unchanged when *n* ranged from 23 to 31, which revealed that the strain hardening exponent *n* has no considerable influence on the ultimate bearing capacity of CFCAT stub columns.

### 3.2. Composite Action of CFCAT Stub Columns

The composite action between aluminum alloy tube and infilled concrete determines the mechanical behaviors of the CFCAT stub columns. Under axial compression, the infilled concrete is confined by the aluminum alloy tube while the tube is enhanced by the infilled concrete as well. From this point of view, the performance of composite columns cannot be only evaluated by the confinement effect exerted by the outer tube (the in-filled concrete expands under compression, and the outer aluminum alloy tubes limit its expansion), the enhancement effect provided by the infilled concrete should be considered either. Only in this way, the compatibility of two components can be evaluated reasonably. To this end, the following indicators were introduced here to explain how the composite action in CFCAT stub columns vary as the changes of design parameters. The radial stress of infilled concrete indicates the lateral compressive stress provided by the aluminum alloy tube on the infilled concrete, the enhancement effect of the infilled concrete can be evaluated by both the axial stress–strain and transverse stress–strain curves of the aluminum alloy tube. In particular, the earlier the axial stress–strain curve intersects with the transverse stress–strain curve, the better the enhancement effect provided by the infilled concrete. These indicators were employed in this study to evaluate the composite action of CFCAT stub columns subjected to axial compression.

#### 3.2.1. Concrete Strength

**Figure 7:**
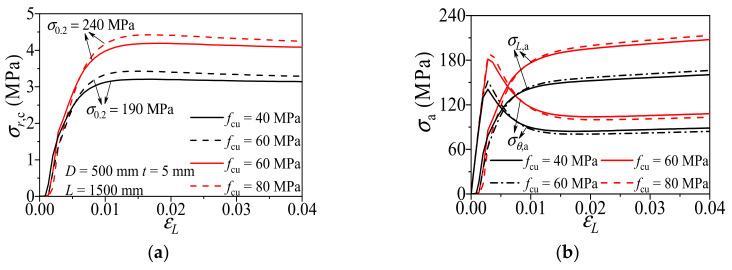
Effect of concrete strength on composite action of CFCAT stub columns: (**a**) radial stress of concrete-axial strain curves; (**b**) stress of aluminum alloy–axial strain curves.

Figure 7 shows the influence of concrete strength (*f*_cu_) on the composite action of CFCAT stub columns. As shown in the figure, the radial stress of concrete, the amplitude and rate of axial stress and transverse stress of aluminum alloy tube increased with the increase of the *f*_cu_. On the other hand, the intersection points of the axial stress–strain curve and transverse stress–strain curve of the aluminum alloy tube of different specimens almost coincide. A conclusion can be drawn here that the higher *f*_cu_ results in a stronger confinement effect in the CFCAT stub columns while no considerable influence on the enhancement.

#### 3.2.2. Yielding Strength of Aluminum Alloy

**Figure 8:**
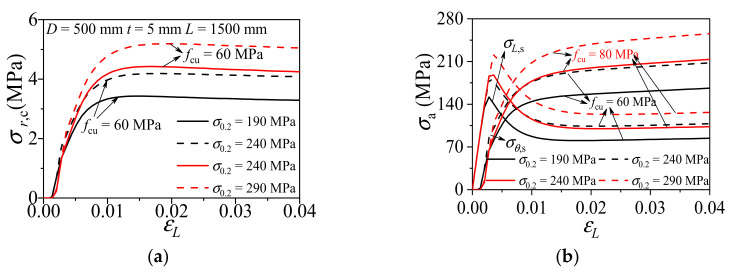
Effect of aluminum alloy yield strength on the composite action of CFCAT stub columns. (**a**) Radial stress of concrete-axial strain curves; (**b**) stress of aluminum alloy–axial strain curves.

Figure 8 shows the influence of the yielding strength of aluminum alloy (*σ*_0.2_) on the composite action of CFCAT specimens. As shown in the figure, the radial stress of concrete increased as the *σ*_0.2_ increased. There is no significant difference in the amplitude and rate of axial stress, but the rate of transverse stress increased rapidly as the increase of *σ*_0.2_. As revealed by those comparisons, the confinement effect was strengthened with the increasement of the yielding strength of the aluminum alloy and the enhancement form infilled concrete was weakened.

#### 3.2.3. Aluminum Ratio

**Figure 9:**
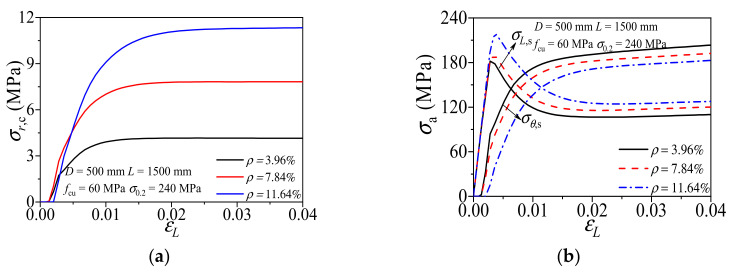
Effect of aluminum ratio on composite action of CFCAT stub columns. (**a**) Radial stress of concrete-axial strain curves; (**b**) Stress of aluminum alloy–axial strain curves.

Figure 9 shows the influence of aluminum ratio (*ρ*) on the composite action of CFCAT specimens. With the same diameter, the numerical results show that the radial stress of concrete increased with the larger aluminum ratio. The intersection point of the axial stress–axial strain curve and transverse stress–axial strain curve was delayed as the increment of aluminum ratio. Hence, the confinement of aluminum alloy tube was strengthened and the enhancement of aluminum alloy tube on core concrete was weakened with the increase of steel ratio.

#### 3.2.4. Elastic Modulus

**Figure 10:**
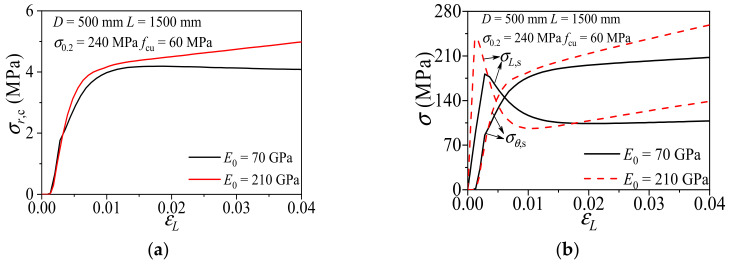
Effect of elastic modulus on the composite action of CFCAT stub columns. (**a**) Radial stress of concrete-axial strain curves; (**b**) stress of aluminum alloy–axial strain curves.

The influences of elastic modulus (*E*_0_) on the composite action of CFCAT specimens is shown in Figure 10. With the same axial strain level, the specimen with higher elastic modulus has the greater concrete radial stress than the low elastic modulus counterpart. When the elastic modulus was larger, the intersection point of axial stress–axial strain curve and transverse stress–axial strain curve appeared earlier. It indicated that the greater the elastic modulus results in the stronger confinement and enhancement effect. The elastic modulus of aluminum alloy is 1/3 to the steel, thus the confinement effect of aluminum alloy tube is weaker than the steel tube.

## 4. Composite Action Model of CFCAT Stub Columns

In the analytical modeling of the axially loaded CFST stub columns, the longitudinal and transverse stress of steel tube at the limit state are the key parameters to determine the load-bearing capacity. This is because of the composite action between steel tube and infilled concrete can be theoretically derived once these parameters were obtained. However, existing studies [42,43,44,45] assume that the steel at the ultimate state of the composite columns satisfy the von Mises yield criterion:

(8) $$\sigma_{l}^{2}+\sigma_{\theta }^{2}-\sigma_{l}\sigma_{\theta }=\sigma_{0.2}^{2}$$

where *σ_l_* and *σ_θ_* represent the longitudinal stress and transverse stress of the metal tube, respectively. This may lead inaccurate prediction due to the following reasons. Firstly, based on the FE simulation, it is found that the metal tube may yield before or after the stub column reached its ultimate state, in this situation, the Equation (6) is invalid. Moreover, the stress component along the thickness direction is neglected, which means the Equation (6) is approximate, especially when the tube wall is thick. To compensate this problem, the presented study extracts the longitudinal and the transverse stress of the aluminum alloy tube when the composite column reached its ultimate state. Since the the aluminum alloy tube was modeled by the solid element, the three-dimensional stress state is considered.

**Figure 11:**
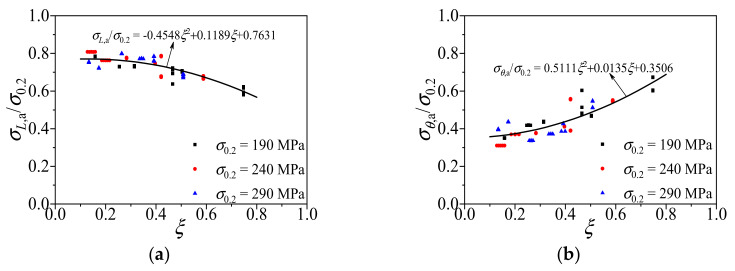
The relationships between stress ratio and confinement factor. (**a**) Relationship between the ratio longitudinal stress to yield stress and the confinement factor; (**b**) relationship between the ratio transverse stress to yield stress and the confinement factor.

The longitudinal stress (*σ_L_*_,a_) and the transverse stress (*σ_θ_*_,a_) of the aluminum alloy tube at mid-height section of all the FE specimens at the ultimate state were captured. It can be found from Figure 11 that the distribution of *σ_L_*_,a_ and *σ_θ_*_,a_ highly correlated to the confinement factor *ξ* (*ξ* = *σ*_0.2_*A*_a_/*f*_c_*A*_c_). Based on the FE analytical results, the numerical model of the composite action in CFCAT stub columns are proposed in Equations (7) and (8) using regression method. It should be noticed here that two independent formula were proposed for *σ_L_*_,a_ and *σ_θ_*_,a_, respectively, instead of using Equation (6).

(9) $$\sigma_{l,a}/\sigma_{0.2}=-0.4548\xi^{2}+0.1189\xi +0.7631$$

(10) $$\sigma_{\theta ,a}/\sigma_{0.2}=0.5111\xi^{2}+0.0135\xi +0.3506$$

## 5. Practical Design Formula for Axial Load-Bearing Capacity of CFCAT Stub Columns

### 5.1. Model Simplification

**Figure 12:**
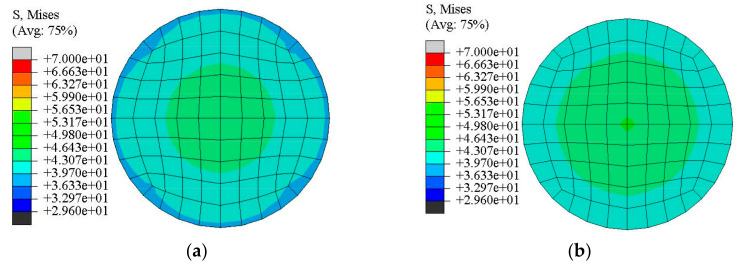
Comparison of stress nephogram at mid-height section for CFCAT and CFCST stub columns. (**a**) CFCAT stub column *f*_cu_ = 40MPa (*f*_c_ = 29.6 MPa); (**b**) CFCST stub column *f*_cu_ = 40MP (*f*_c_ = 29.6 MPa).

**Figure 13:**
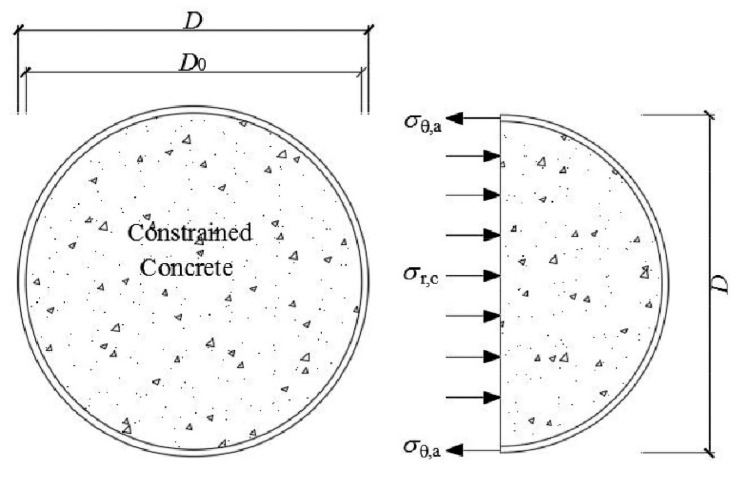
Simplified stress distribution model at the mid-height section of CFCAT stub columns.

The infilled concrete nephogram at mid-height section for CFCAT and CFCST stub columns at the limit state obtained by the FE analysis were compared in Figure 12. The axial stresses of the confined concrete in CFCAT and CFCST stub columns were expressed in shades of color. The figures indicate that the confinement effect occurred at the whole infilled concrete cross-section of both types of columns, and it is weaker in CFCAT stub column than that of steel tube counterparts. Based on Figure 12, the stress condition of CFCAT stub columns at limit state could be simplified as shown in Figure 13. The simplification rationally follows the stress distribution and superposition theory when the composite column reached the ultimate limit state. In the figure, *D* and *D*_0_ are the diameter and the inner diameter of aluminum alloy tube, respectively; *A*_c_ and *A*_a_ are the dimension of infilled concrete and aluminum alloy tube, respectively; *σ_r_*_,c_ and *σ_θ_*_,a_ is the radial stress of infilled concrete and the transverse stress of aluminum alloy tube, respectively.

Based on the force equilibrium conditions shown in Figure 11, the following equation can be derived as:

(11) $$\sigma_{r,c}=\frac{\rho }{2(1-\rho )}\sigma_{\theta ,a}$$

(12) $$\rho =\frac{D^{2}-D_{0}{}^{2}}{D^{2}}$$

According to the previous study about the confined concrete [46], the relationship of axial stress and the radial stress considering the confinement effect of infilled concrete can be expressed as:

(13) $$\sigma_{L,c}=f_{c}+3.4\sigma_{r,c}$$

### 5.2. Derivation of Design Formula of CFCAT Stub Columns

According to the static equilibrium condition of cross-section, the ultimate bearing capacity of CFCAT stub columns can be expressed as:

(14) $$N_{u}=\sigma_{L,c}A_{c}+\sigma_{L,a}A_{a}$$

Substituting Equations (7)–(11) into Equation (12), the ultimate bearing capacity (*N*_u1_) of CFAST stub columns can be expressed as:

(15) $$N_{u1}=f_{c}A_{c}+(0.4141\xi^{2}+0.1419\xi +1.3591)\sigma_{0.2}A_{a}$$

**Figure 14:**
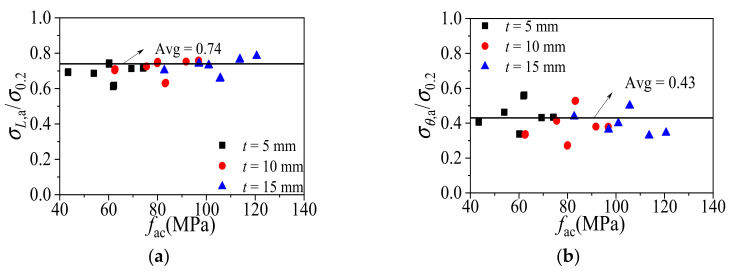
Axial stress and transverse stress of CFCAT stub columns. (**a**) Average ratio of longitudinal stress to yield stress; (**b**) average ratio of transverse stress to yield stress.

To simplify the calculation process, the average ratio of *σ_L_*_,a_ – *σ*_0.2_ and *σ_θ_*_,a_ – *σ*_0.2_ can be employed instead of the Equations (7) and (8). Figure 14 shows the calculated relationships between the *σ_L_*_,a_–*σ*_0.2_ ratio, *σ_θ_*_,a_–*σ*_0.2_ ratio and the specimen’s ultimate strength. For CFCAT stub columns, the average ratio of *σ_L_*_,a_–*σ*_0.2_ was 0.74, greater than 0.69 of the CFCST stub columns; the average ratio of *σ_θ_*_,a_–*σ*_0.2_ was 0.43, smaller than 0.55 of the CFCST stub columns [32]. The relationship between *σ_L_*_,a_–*σ*_0.2_ and *σ_θ_*_,a_–*σ*_0.2_ can be taken as:

(16) $$\sigma_{L,a}=0.74\sigma_{0.2}$$

(17) $$\sigma_{\theta ,a}=0.43\sigma_{0.2}$$

Substituting Equations (9)–(11) and Equations (14) and (15) into Equation (12), the ultimate axial bearing capacity (*N*_u2_) can be derived as:

(18) $$N_{u2}=f_{c}A_{c}+K\sigma_{0.2}A_{a}$$

where *K* is the enhancement factor [47] which reflects the enhancement effect provided by the infilled concrete to the outer tube. For CFCAT stub columns, *K* is 1.47, which is lower than 1.62 of CFCST stub columns [32].

### 5.3. Formula Validation

**Figure 15:**
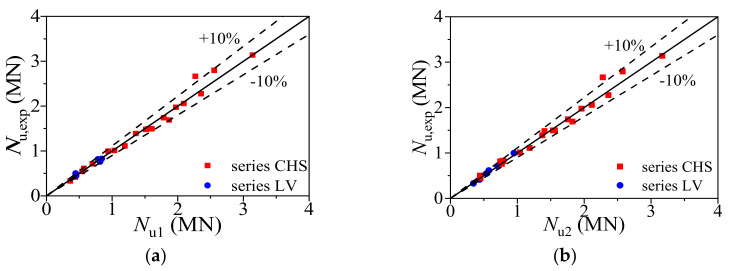
Comparison of ultimate bearing capacity. (**a**) Comparison of the ultimate bearing capacities obtained from test results and Equation (15; (**b**) comparison of the ultimate bearing capacities obtained from test results and Equation (18).

The ultimate bearing capacities calculated from Equation (15) (*N*_u1_) and Equation (18) (*N*_u2_) were compared with collected test results (*N*_u,exp_) and previously performed FE analysis results (*N*_u,FE_), as shown in Figure 15. The average ratios of *N*_u,exp_ to *N*_u1_ and *N*_u2_ are 1.004 and 0.991 with the dispersion coefficients of 0.049 and 0.055, respectively. The consequences of Equations (15) and (18) showed that there is nearly no difference using these two formulas to calculate the ultimate bearing capacity of the CFCAT stub columns. The Equation (18) has a relatively safe calculation result and the simple form, which is beneficial to the application in practical engineering.

**Figure 16:**
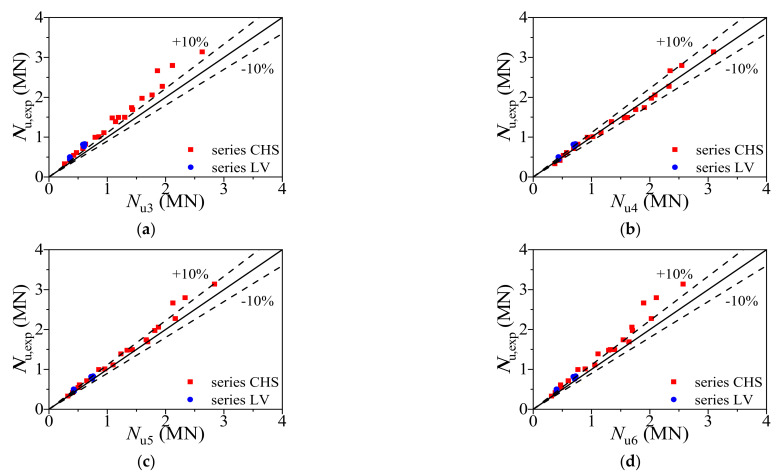
Comparison of ultimate bearing capacity. (**a**) Comparison of the ultimate bearing capacities obtained from test results and Equation (19); (**b**) comparison of the ultimate bearing capacities obtained from test results and Equation (20); (**c**) comparison of the ultimate bearing capacities obtained from test results and Equation (21); (**d**) comparison of the ultimate bearing capacities obtained from test results and Equation (22).

Table 3 showed the formulas of axial bearing capacity provided by different well-known design codes. The results obtained from those formulas are compared with test results in Table 4. The average ratio of *N*_u,exp_ to *N*_u3_, *N*_u4_, *N*_u5_ and *N*_u6_ is 0.799, 0.986, 0.921 and 0.864 with the corresponding dispersion coefficient of 0.061, 0.077, 0.056 and 0.076, respectively. The ultimate bearing capacities calculated from Equation (17) (*N*_u4_) to Equation (20) (*N*_u6_) were compared with collected test results (*N*_u,exp_), as shown in Figure 16. As a result, the proposed formula (Equation (12)) has higher accuracy in predicting the axial bearing capacity of CFCAT stub columns.

## 6. Conclusions

This paper investigated the confinement effects of the CFCAT stub columns under axial compression. The main contents and conclusions are as follows:

- A fine-meshed finite 3D solid element model of CFCAT under axial compression was established based on the tri-axial plastic-damage constitutive model of concrete and elastoplastic constitutive model of the aluminum alloy. The FE analytical results coincide well with the experimental results.
- Based on the validated FE modeling technique, 90 full-scale FE models were established for parametric study. The numerical results revealed that the higher aluminum alloy strength and ratio lead to a better confinement effect and a weakened enhancement effect. As a result, the compatibility of the strength and dimension of the aluminum alloy and concrete should be noticed in the design, rather than only seeking better confinement.
- Regression models of the longitudinal stress and transverse stress of the aluminum alloy tube at the ultimate state of the columns were proposed, respectively. This model considered the three-dimensional stress state of the outer tube and is a more authentic expression when the column reached its ultimate state.
- A design formula was proposed to estimate the ultimate bearing capacity of CFCAT stub columns under axial compression. An enhancement factor which reflects the level of composite action of CFCAT stub columns is calculated. The obtained value of 1.47, which is smaller than the confinement coefficient of 1.62 of CFCST stub columns. The proposed formula that was evaluated has a higher accuracy compared with some current design methods.

## Figures and Tables

**Table 1:** Comparison of collected test results and FE analysis results.

Specimens	Ref.	D×t×*L* (mm)	*E*_0_ (MPa)	*σ*_0.2_ (MPa)	*n*	*f*_cu_ (MPa)	N_u,exp_ (kN)	N_u,FE_ (kN)	N_u,exp_/N_u,FE_
CHS4-C40	[7]	76.1 × 2.06 × 228	64,900	237.0	23.70	56.0	329.9	383.5	0.860
CHS4-C70		76.0 × 2.06 × 228	64,900	237.0	23.70	80.2	415.7	450.2	0.923
CHS4-C100		76.1 × 2.05 × 228	64,900	237.0	23.70	114.0	611.4	599.5	1.020
CHS5-C40		99.7 × 2.02 × 300	65,600	244.3	24.43	56.0	543.6	557.5	0.975
CHS5-C70		99.8 × 2.06 × 300	65,600	244.3	24.43	80.2	712.0	692.8	1.028
CHS5-C100		100 × 2.05 × 300	65,600	244.3	24.43	114.0	995.8	947.0	1.052
CHS6-C40		119.8 × 2.49 × 360	66,500	253.1	25.31	56.0	822.8	826.3	0.996
CHS6-C70		120 × 2.55 × 360	66,500	253.1	25.31	80.2	1010.3	1023.9	0.987
CHS6-C100		119.6 × 2.48 × 360	66,500	253.1	25.31	114.0	1388.7	1377.2	1.008
CHS7-C40		150.1 × 2.53 × 450	64,900	267.9	26.79	56.0	1111.1	1212.2	0.917
CHS7-C70		150.1 × 2.54 × 451	64,900	267.9	26.79	80.2	1496.4	1520.4	0.984
CHS7-C100		149.9 × 2.53 × 450	64,900	267.9	26.79	114.0	2057.8	2108.4	0.976
CHS8-C40		150.2 × 5.03 × 228	65,800	216.9	21.69	56.0	1481.9	1432.7	1.034
CHS8-C70		150.2 × 5.04 × 450	65,800	216.9	21.69	80.2	1740.6	1661.9	1.047
CHS8-C100		150.2 × 5.03 × 450	65,800	216.9	21.69	114.0	2666.1	2171.0	1.228
CHS9-C40		160.1 × 4.03 × 480	66,600	254.2	25.42	56.0	1494.1	1544.9	0.967
CHS9-C70		160.5 × 4.07 × 480	66,600	254.2	25.42	80.2	1974.4	1928.0	1.024
CHS9-C100		160.5 × 4.06 × 480	66,600	254.2	25.42	114.0	2797.3	2551.6	1.096
CHS10-C40		180.0 × 3.71 × 540	68,700	264.9	26.49	56.0	1690.2	1913.1	0.883
CHS10-C70		180.4 × 3.69 × 540	68,700	264.9	26.49	80.2	2274.2	2339.5	0.972
CHS10-C100		180.5 × 3.75 × 540	68,700	264.9	26.49	114.0	3139.2	3172.0	0.990
LV100-1	[41]	100 × 2 × 300	89,752	186.4	18.64	50.08	443.6	483.0	0.918
LV100-2		100 × 2 × 300	86,956	187.8	18.78	50.08	448.85	485.3	0.923
LV100-3		100 × 2 × 300	90,878	182.0	18.20	50.08	502.3	479.5	1.048
LV120-1		120 × 4 × 360	105,661	170.8	17.08	50.08	815.9	811.3	1.005
LV120-2		120 × 4 × 360	92,593	188.8	18.88	50.08	829.7	862.7	0.962
LV120-3		120 × 4 × 360	91,863	181.1	18.11	50.08	761.5	839.6	0.907
Mean									0.996
Cov									0.073

**Table 2:** Material and geometric properties of specimens for FE parametric study.

*D* (mm)	*L* (mm)	*t* (mm)	*n*	*f*_cu_ (MPa)	*σ*_0.2_ (MPa)
500	1500	5	23–31	40, 60	190
		5	23–31	60, 80	240
		5	23–31	80, 100	290
		10	23–31	40, 60	190
		10	23–31	60, 80	240
		10	23–31	80, 100	290
		15	23–31	40, 60	190
		15	23–31	60, 80	240
		15	23–31	80, 100	290

**Table 3:** Summary of available formulas in well-known national codes.

No.	Ref.	Formula	Limitations
Equation (19)	ACI 318 [18]	$N_{u3}=A_{a}\sigma_{0.2}+0.85A_{c}f_{c}{}^{\prime }$	-
Equation (20)	Zhou, F. [21]	$\begin{array}{l} N_{u4}=A_{a}\sigma_{0.2}+0.85A_{c}{f^{\prime }}_{c}+\eta A_{c}\sigma_{0.2}{}_{}\\ \left\{\begin{array}{l} \eta =0.31-0.0055\frac{D}{t},\;9\leq \frac{D}{t}\leq 50\left(1-0.377\frac{{f^{\prime }}_{c}}{\sigma_{0.2}}\right)\\ \eta =0.045-0.0002\frac{D}{t}+0.1\left(\frac{{f^{\prime }}_{c}}{\sigma_{0.2}}\right),\;50\left(1-0.377\frac{{f^{\prime }}_{c}}{\sigma_{0.2}}\right)<\frac{D}{t}\leq 160 \end{array}\right. \end{array}$	$\begin{array}{l} f_{c}{}^{\prime }\leq 106\mathrm{MPa}\\ 9\leq \frac{D}{t}\leq 160 \end{array}$
Equation (21)	Wang, F.C. [22]	$\begin{array}{l} N_{u5}=f_{\mathrm{acy}}(A_{a}+A_{c})\\ \frac{f_{\mathrm{acy}}}{f_{\mathrm{ck}}}=1.14+1.02\xi \\ f_{\mathrm{ck}}=0.67f_{\mathrm{cu}},\;\xi =\frac{A_{a}\sigma_{0.2}}{A_{c}f_{\mathrm{ck}}} \end{array}$	0.03≤ρ≤0.2
Equation (22)	Zha, X.X. [48]	$\begin{array}{l} N_{u6}=f_{\mathrm{acy}}(A_{a}+A_{c})\\ f_{\mathrm{acy}}=\frac{1+1.5(k\xi )}{1+B\xi }f_{\mathrm{ck}}\\ k=\frac{2}{3}k_{e}+\frac{1}{3},\;k_{e}=\frac{412\alpha +1.82}{415\alpha +5.46},\\ B=\frac{f_{\mathrm{ck}}}{\sigma_{0.2}},\;\xi =\frac{A_{a}\sigma_{0.2}}{A_{c}f_{\mathrm{ck}}},\;\alpha =\frac{A_{a}}{A_{c}} \end{array}$	-

**Table 4:** Comparison between experimental and predicted result using different design methods.

Specimens	Ref.	*N*_u,exp_ (kN)	*N*_u2_ (kN)	*N*_u3_ (kN)	*N*_u4_ (kN)	*N*_u5_ (kN)	*N*_u6_ (kN)	*N*_u,exp_/ *N*_u2_	*N*_u,exp_/ *N*_u3_	*N*_u,exp_/ *N*_u4_	*N*_u,exp_/ *N*_u5_	*N*_u,exp_/ *N*_u6_
CHS4-C40	[7]	329.9	345.1	286.6	371.3	323.9	314.5	1.046	0.814	1.126	0.982	0.953
CHS4-C70		415.7	436.8	383.8	458.3	407.0	379.6	1.051	0.855	1.102	0.979	0.913
CHS4-C100		611.4	574.7	515.0	574.8	524.6	471.9	0.940	0.773	0.940	0.858	0.772
CHS5-C40		543.6	537.2	457.1	518.7	501.5	482.3	0.988	0.782	0.954	0.922	0.887
CHS5-C70		712	705.8	633.7	695.7	650.3	603.5	0.991	0.819	0.977	0.913	0.848
CHS5-C100		995.8	951.3	867.3	929.5	854.4	768.1	0.955	0.796	0.933	0.858	0.771
CHS6-C40		822.8	794.7	672.6	765.3	739.7	715.0	0.966	0.761	0.930	0.899	0.869
CHS6-C70		1010.3	1040.3	929.2	1022.5	957.3	892.3	1.030	0.848	1.012	0.947	0.883
CHS6-C100		1388.7	1375.2	1250.1	1342.4	1234.0	1112.9	0.990	0.823	0.967	0.889	0.801
CHS7-C40		1111.1	1185.2	1016.9	1163.5	1099.6	1056.0	1.067	0.849	1.047	0.990	0.950
CHS7-C70		1496.4	1563.3	1416.3	1563.0	1427.9	1325.4	1.045	0.869	1.045	0.954	0.886
CHS7-C100		2057.8	2114.6	1940.7	2087.0	1878.5	1693.7	1.028	0.860	1.014	0.913	0.823
CHS8-C40		1481.9	1406.5	1153.5	1571.8	1340.2	1294.2	0.949	0.732	1.061	0.904	0.873
CHS8-C70		1740.6	1759.2	1526.1	1906.3	1668.8	1545.3	1.011	0.815	1.095	0.959	0.888
CHS8-C100		2666.1	2279.1	2020.5	2347.6	2124.7	1893.3	0.855	0.698	0.881	0.797	0.710
CHS9-C40		1494.1	1533.0	1274.3	1615.1	1428.4	1394.6	1.026	0.798	1.081	0.956	0.933
CHS9-C70		1974.4	1961.0	1723.5	2026.8	1813.9	1701.9	0.993	0.808	1.027	0.919	0.862
CHS9-C100		2797.3	2575.5	2307.8	2547.2	2334.5	2113.1	0.921	0.757	0.911	0.835	0.755
CHS10-C40		1690.2	1824.1	1539.1	1757.7	1691.5	1642.6	1.079	0.849	1.040	1.001	0.972
CHS10-C70		2274.2	2362.8	2109.6	2328.8	2166.4	2025.5	1.039	0.855	1.024	0.953	0.891
CHS10-C100		3139.2	3169.5	2872.6	3092.7	2839.1	2570.5	1.010	0.837	0.985	0.904	0.819
LV100-1	[41]	443.6	448.2	390.2	438.2	428.4	404.8	1.010	0.816	0.988	0.966	0.913
LV100-2		448.85	449.4	391.0	439.4	429.4	406.0	1.001	0.808	0.979	0.957	0.905
LV100-3		502.3	444.2	387.5	434.4	425.4	401.0	0.884	0.715	0.865	0.847	0.798
LV120-1		815.9	746.3	623.8	690.5	725.5	689.8	0.915	0.717	0.846	0.889	0.845
LV120-2		829.7	784.8	650.0	723.6	756.2	727.5	0.946	0.737	0.872	0.911	0.877
LV120-3		761.5	768.3	638.8	709.5	743.1	711.4	1.009	0.788	0.932	0.976	0.934
Mean								0.991	0.799	0.986	0.921	0.864
Cov								0.055	0.061	0.077	0.056	0.076

## Data Availability

No new data were created or analyzed in this study. Data sharing is not applicable to this article.
